# Locomotive Syndrome Digital Therapeutics Provided via a Smartphone App: Proof-of-Concept Single-Group Trial Study

**DOI:** 10.2196/86174

**Published:** 2026-03-13

**Authors:** Tatsuru Sonobe, Itaru Ogawa, Takahiro Seki, Kosuke Watanabe, Yota Kaneko, Takeru Yokota, Taro Mawatari, Satoru Harada, Yasumichi Kadowaki, Youhei Takenaka, Yoshihiro Matsumoto

**Affiliations:** 1 Department of Orthopaedic Surgery, School of Medicine, Fukushima Medical University, 1 Hikariga-oka, Fukushima, 960-1295, Japan, 81 24-547-1276, 81 24-548-5505; 2 Department of Orthopaedic Surgery, KKR Hamanomachi Hospital, Fukuoka, Japan; 3 Future Corporation, Tokyo, Japan

**Keywords:** locomotive syndrome, LS, digital therapeutic, DTx, locomotion training, LT, Timed Up and Go test, TUG test, 25-question Geriatric Locomotive Function Scale, 25-GLFS, Behavioral Regulation in Exercise Questionnaire-3, BREQ-3

## Abstract

**Background:**

Individuals with locomotive syndrome (LS) have muscle weakness and reduced motor function due to musculoskeletal disorders that cause reduced mobility and physical function. In Japan, musculoskeletal disorders are the most common reason for requiring home support or nursing care, highlighting the need for preventing and ameliorating LS. Middle-aged and older adults sometimes encounter difficulty making a habit of exercise therapy (the mainstay of LS treatment).

**Objective:**

We investigated whether digital therapeutics (DTx) can prevent or ameliorate LS in middle-aged and older adults.

**Methods:**

We conducted a prospective, longitudinal, nonrandomized, single-group study of Japanese aged 40 years and older who were eligible for LS checkups (N=47). Each participant underwent an 8-week locomotion training intervention based on DTx supervised by medical staff. We objectively assessed the participants’ subjective and objective motor abilities and motor awareness with the Timed Up and Go (TUG) test, 25-question Geriatric Locomotive Function Scale (GLFS-25), and Behavioral Regulation in Exercise Questionnaire-3 at baseline (before the DTx), an interim point (4 wk after the DTx initiation), and a final evaluation (8 wk post-DTx initiation). We compared the scores of the 3 tests at the 3 time points as dependent variables in a 3-factor ANOVA with Bonferroni correction (significance defined as 0.05/3=0.0167).

**Results:**

No increase in amotivation to exercise or refusal to exercise was observed. Significant improvements at 8 weeks versus the baseline were observed in the TUG scores (baseline: 9.0, 95% CI 8.4‐9.6; 8 wk: 7.5, 95% CI 7.1‐8.0; *P*=.001) and GLFS-25 results (baseline: 18.7, 95% CI 14.5‐22.8; 8 wk: 11.7, 95% CI 8.8‐14.7; *P*=.004). The Behavioral Regulation in Exercise Questionnaire-3 and its subscale data did not differ significantly at any assessment time point.

**Conclusions:**

These results indicate that an 8-week locomotive training intervention using DTx significantly improved middle-aged and older adults’ TUG and GLFS-25 scores and will help prevent and ameliorate LS and establish better exercise habits among them.

## Introduction

The Japanese Orthopaedic Association (JOA) proposed the concept of locomotive syndrome (LS) in 2007 [1]. The term “LS” refers to a condition in which a musculoskeletal disorder results in muscle weakness and motor weakness, leading to reduced mobility and physical function [1]. The development of LS causes a requirement for long-term care [2]. According to the 2022 Comprehensive Survey of Living Conditions in Japan, the leading cause requiring nursing care or support was musculoskeletal disorders at 26.3%, followed by falls and fractures at 13.9% and joint disorders at 10.2% [3]. The JOA recommends the early detection of a decline in motor function based on locomotive syndrome checks (LSCs), in part to prevent the requirement of assistance or care [4]. LSCs were scored as follows: 1 point (cannot put on a sock for 1 foot), 2 points (stumbles in their home), 3 points (needs a handrail to climb stairs), 4 points (difficulty using a vacuum cleaner at home), 5 points (difficulty carrying parcels weighing >2 kg), 6 points (difficulty walking continuously for >15 min), and 7 points (cannot cross a crosswalk with a green light) [4]. The JOA also advocates locomotion training (LT), which involves standing on 1 leg and squatting, to prevent LS and lessen existing LS [5]. Currently, the risk of developing LS is assessed based on three items: (1) a stand-up test, (2) a 2-step test, and (3) the 25-question Geriatric Locomotive Function Scale (GLFS-25) [5-7].

Exercises such as LT are effective for preventing and mitigating LS [5], but it is difficult for many middle-aged and older adults to start or continue an exercise routine. Previous studies have reported that the average adherence rate for older adults continuing a prescribed home exercise program for 6 weeks was 65% [8]. As one solution, we advocate an approach to the prevention and mitigation of LS in middle-aged and older adults who meet the criteria for LS or qualify for LSCs using IT devices.

Apart from the uses of IT in the medical field, such as electronic medical records, telemedicine, and surgical robots, the uses of mobile devices for medical procedures and support are called mobile health [9]. Digital therapeutics (DTx), defined by the Digital Therapeutics Alliance as “evidence-based therapeutic interventions driven by high-quality software programs to prevent, manage, or treat a medical disorder or disease” [10], is an area of mobile health that is receiving particular attention [11]. A variety of DTx apps have been approved by health insurance programs in some countries, including those for diabetes, hypertension self-management, and smoking cessation support [12-14]. Regarding the effects of DTx on the musculoskeletal system, the effectiveness of DTx in musculoskeletal diseases has been reported for patients with pain associated with musculoskeletal diseases and osteoarthritis [15,16]. DTx has been shown to be effective in reducing musculoskeletal pain, improving function in patients with osteoarthritis, and increasing self-management abilities. However, there have been no reports on the use of DTx intervention for LS. If DTx intervention can establish exercise habits and lead to the prevention and improvement of LS, it may lead to a reduction in the number of people in Japan who require nursing care or support due to musculoskeletal diseases. We conducted this study to determine whether this use of DTx via a smartphone app can lead to behavioral change in middle-aged and older people and prevent or ameliorate their LS.

## Methods

### Participants

The details of the research protocol and participant recruitment have been published [17]. The study population was 47 Japanese participants aged more than 40 years who were eligible for the LSCs. The study’s inclusion criteria were as follows: individuals aged 40 years and older who generally begin to experience a decline in lower-extremity muscle strength [18] and met any of the LSC criteria. Additional inclusion criteria were: a score of 3 points or more on the Mini-Cog. The Mini-Cog is a screening test for cognitive function assessment combining 3-word immediate recall, delayed recall, and clock drawing. A score of less than 3 points indicates suspected dementia, with reported sensitivity of 76% to 99% and specificity of 83% to 93% [19,20]. The ability to use smartphone apps and being able to provide informed consent for study participation were additional requirements. Exclusion criteria were difficulty using smartphone apps due to cognitive decline or visual or hearing impairment, withdrawal during the study period, and withdrawal during the study period due to hospitalization or accidents. The participants’ demographic information was recorded, including age, gender, musculoskeletal disorders considered potential causes of LS, and underlying conditions corresponding to LSCs.

### DTx Intervention

**Figure 1.**
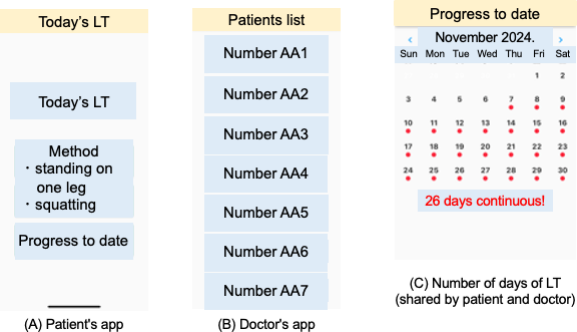
Screenshots of the app. (A) The app for the patients or participants has a simple design and enabled them to perform the locomotion training (LT). (B) The physician’s app can check each patient’s LT status based on the ID allocated to that patient. (C) The number of days of LT is reflected on the same screen for both the patient and the physician.

We have created a smartphone app for middle-aged and older people who have no exercise habits and whose physical functions have declined or are suspected to be declining. The smartphone app used by the participants is a new prototype app created for this research field. We provided a smartphone equipped with a communication environment with the app already downloaded to each participant. This smartphone app has functions that support the implementation of 2 types of LT with the use of video and audio. The LT app is simple to use and is set up so that by touching the screen, a voice announcement is played, and the user can perform 2 LT programs: the 1-leg stand program and the squat program. After the completion of the day’s LT, the participant touched the screen to check the LT record on both the participant’s smartphone and his or her physician’s smartphone (Figure 1). The smartphone app automatically records the number of days of LT.

Participants received direct instruction on how to use the app and perform LT from their attending physician during an outpatient visit at the medical institution involved in this study. The prior instruction took an average of 10 minutes. Furthermore, after the instruction, participants were able to use the DTx without any problems. Although there were several inquiries regarding its use, participants contacted the research staff directly by phone, and all inquiries were resolved over the phone. Each participant was scheduled for the daily LT intervention for 8 weeks. A single LT session consisted of a program of standing on 1 leg for 60 seconds (or <60 s if that was the participant’s best effort) plus 5 squats, performed 3 times per day, as recommended by the JOA for the prevention and improvement of LS [5]. For participants with weak legs and/or backs who would find it difficult to do the regular LT, the physician responsible for explaining the DTx advised that they stand on 1 leg while supporting their body with their hands or fingers on a desk or table, or that they sit on a chair and stand up by putting their hands on a desk or table instead of doing squats, or that they reduce the number of times they do each LT from 3 times to 1 time [21].

The advantage of this system is that healthcare providers can check the progress of their patients’ LT remotely, enabling them to prepare feedback in advance and freeing up time. It also provides patients with the reassurance that they are exercising under the support of medical staff.

### Study Protocol

Briefly, the participants’ objective mobility was assessed using the Timed Up and Go (TUG) test score; their subjective mobility was evaluated with the GLFS-25, and the changes in exercise-related motivation were investigated based on the Behavioral Regulation in Exercise Questionnaire-3 (BREQ-3). The GLFS25 is a questionnaire that assesses physical pain and difficulties in daily life using 25 questions. Each question is rated on a scale of 0 to 4, resulting in a total score of 0 to 100 [5-7]. The self-determination theory (SDT) [22] is widely applied as a theoretical foundation for evaluating the effectiveness of behavioral change in participants. The BREQ-3 is designed based on SDT and assesses motivation for exercise with 18 questions in 6 areas: intrinsic motivation, integrative adjustment, identification adjustment, incorporative adjustment, external adjustment, and demotivation [23]. Previous studies have adopted the BREQ-3 as an indicator for evaluating behavioral change [24]. In this study, the impact of DTx on motivation for exercise and exercise spontaneity was also evaluated using the BREQ-3. The participants underwent the TUG test, GLFS-25, and BREQ-3 measurements at baseline (before the DTx intervention was started), and at 4 weeks (intermediate evaluation) and 8 weeks (final evaluation) after the start of the DTx intervention. In addition, the implementation status and compliance rate of the LT during the survey period were analyzed. This study primarily aimed to explore the efficacy, feasibility, and behavioral impact of a DTx intervention for LT. Accordingly, a single-group pre-post comparative design was adopted to efficiently evaluate within-subject changes in objective mobility, subjective mobility, and exercise-related motivation. Outcome measures and the intervention duration were set at a feasible timeframe capable of detecting clinical effects while minimizing participant burden.

### Statistical Analyses

Continuous data are summarized as the mean and SD, and dichotomous or categorical data are presented as proportions. As the main study outcomes, the TUG test and GLFS-25 results were measured at 0 weeks before the DTx intervention (baseline) and at 4 and 8 weeks after the daily DTx intervention was started. TUG and GLFS-25 data at each assessment point were compared using ANOVA. Furthermore, the Pearson correlation coefficients were calculated between the participants’ baseline TUG scores and the difference in their TUG scores (Δ) after 8 weeks of DTx in each case. Similarly, the Pearson correlation coefficients were calculated for the difference between baseline and post-8 weeks DTx GLFS-25 scores. As a secondary outcome measure in this study, participants’ BREQ-3 scores obtained at 3 time points were also compared using the Wilcoxon signed-rank test. The *P* values after Bonferroni correction that were <.05/3=0.0167 were considered significant. All analyses were conducted using JMP PRO 16 (SAS Institute).

The reported minimal clinically important difference (MCID) in the TUG test among patients with degenerative disc disease is 3.4 (SD 5.0) seconds [25]. Considering that finding, we set the MCID at 3.0 seconds for the present investigation. The required sample size was calculated using G^*^power (version 3.1.9.7; Heinrich-Heine-Universität Düsseldorf).[26]. The corresponding 2-tailed *t* test was selected, and the effect size was calculated as 0.6 based on a mean difference of 3.0 and a SD of difference of 5.0. Assuming an α-value of .05 and .80 as the power (1−β), the required estimated sample size was 24. When taking into account both the possibility that the SD is smaller than those in the previous studies and the cases of dropout, we concluded that the study had the required sample size.

### Ethical Considerations

The Fukushima Medical University Ethics Review Committee provided ethical approval for this study (REC 2023‐196). All study methods followed the relevant guidelines and regulations, and written informed consent was obtained from participants. This study was preregistered as a trial registration (University Hospital Medical Information Network Clinical Trial Registry UMIN000053922). Data was deidentified to protect participant information. Participants were not paid compensation and cooperated voluntarily.

## Results

### Recruitment Results Flowchart

**Figure 2.**
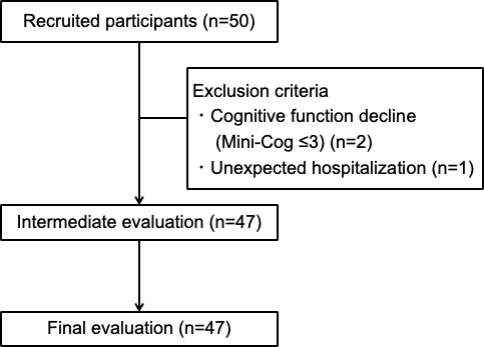
Flowchart of participants. Two individuals with cognitive impairment and one who was unexpectedly hospitalized were excluded from the study. After the start of the study, there were no dropouts, and 47 participants were followed up until the intermediate and final evaluations.

Figure 2 illustrates the flow of participants over the course of the study. Two participants for whom cognitive function decline was observed and 1 participant who was unexpectedly hospitalized were excluded from the study. There were no dropouts after the start of the study, and all participants completed the final evaluation.

### Participants’ Characteristics

The characteristics of the 47 participants are summarized in Table 1. Most of the participants (55.3%, n=26) were aged under 65 years, and 87.2% of the participants were women. All of the participants had preserved cognitive function. The most common cause of LS in this population was knee osteoarthritis (51.1%).

**Table 1.** The participants’ characteristics (N=47).

Characteristics	Value
Days of LT^a^, mean (SD, 95% CI)	52.1 (5.9, 50.4‐53.8)
Age (y), mean (SD, 95% CI)	63.5 (10.1, 60.5‐66.5)
<65, n (%)	26 (55.3)
65‐74, n (%)	13 (27.7)
≥75, n (%)	8 (17)
Sex, n (%)	
Male	6 (12.8)
Female	41 (87.2)
Mini-Cog score (points), n (%)	
4	11 (23.4)
5	36 (76.6)
Disease causing LS^b^, n (%)	
Knee osteoarthritis	24 (51.1)
Hip osteoarthritis	5 (10.6)
Lumbar spondylosis	14 (29.7)
Hip fracture	2 (4.3)
Ankle osteoarthritis	2 (4.3)

a LT: locomotion training.

b LS: locomotive syndrome.

### Primary Outcome Measures

#### TUG Test

The changes in the participants’ TUG scores from baseline to the end of the 8-week intervention are depicted in Table 2. Notably, with the DTx, all but 1 of the 47 participants achieved an improvement in their TUG score. The TUG score at baseline was 9.0 (95% CI 8.4‐9.6), reduced significantly to 8.0 (95% CI 7.6‐8.5) at 4 weeks (*P*=.01). Furthermore, compared to baseline, the TUG score significantly decreased to 7.5 (95% CI 7.1‐8.0) at week 8 (*P*=.001).

**Table 2.** The participants’ Timed Up and Go (TUG) test data at baseline, 4 weeks, and 8 weeks^a^.

	TUG, mean (SD, 95% CI)	Adjusted *P* value^b^
Baseline	9.0 (2.1, 8.4‐9.6)	—^c^
4 wk	8.0 (1.5, 7.6‐8.5)	.01^d^
8 wk	7.5 (1.5, 7.1‐8.0)	.001^d^

a The data are the mean, n (%), and ANOVA (95% CI) results.

b Adjusted *P* values represent comparisons with baseline.

c Not applicable.

d *P*<.05.

**Figure 3.**
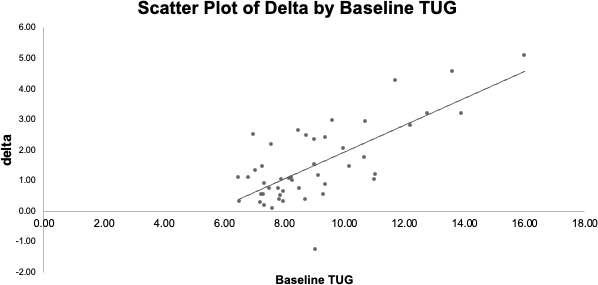
The correlation between the baseline Timed Up and Go (TUG) score and the difference in the TUG score after 8 weeks of digital therapeutics (DTx) in each participant’s case. The TUG score at baseline was positively correlated with the improvement in the TUG score obtained after 8 weeks of DTx (*r*=0.728; *P*<.001).

We next examined the correlation. The analyses revealed that the baseline TUG score was significantly positively correlated with the improvement in TUG score obtained after 8 weeks of DTx (Pearson correlation coefficient, ie, *r*=0.728; *P*<.001; Figure 3).

#### 25-Question Geriatric Locomotive Function Scale

The results on this scale revealed that the GLFS-25 score improved in 43 of the 47 patients, with mean scores of 18.7 (95% CI 14.5‐22.8) points at baseline, 12.9 (95% CI 10.4‐15.4) points at 4 weeks, and 11.7 (95% CI 8.8‐14.7) points at 8 weeks. The GLFS-25 at 8 weeks was significantly lower than the baseline values (*P*=.004; Table 3).

**Table 3.** The participants’ 25-question Geriatric Locomotive Function Scale (GLFS-25) scores at baseline, 4 weeks, and 8 weeks of digital therapeutics (DTx)^a^.

	GLFS-25, mean (SD, 95% CI)	Adjusted *P* value^b^
Baseline	18.7 (14.2, 14.5‐22.8)	—^c^
4 wk	12.9 (8.4, 10.4‐15.4)	.02^d^
8 wk	11.7 (10.1, 8.8‐14.7)	.004^d^

a The data are the mean, n (%), and ANOVA (95% CI) results.

b Adjusted *P* values represent comparisons with baseline.

c Not applicable.

d *P*<.05.

**Figure 4.**
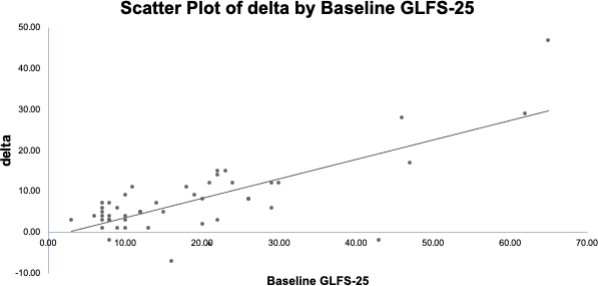
The correlation between the baseline 25-question Geriatric Locomotive Function Scale (GLFS-25) score and the difference in the GLFS-25 score after 8 weeks of digital therapeutics (DTx) in each participant’s case. A high correlation was observed, indicating that participants with higher initial GLFS-25 scores showed greater improvement after the 8-week intervention (*r*=0.749; *P*<.001).

When the correlation between the Δ in the GLFS-25 score from baseline to 8 weeks and the initial value was examined, a higher correlation was observed, with the participants who had higher initial GLFS-25 scores showing greater improvement at 8 weeks (*r*=0.749; *P*<.001; Figure 4).

### Secondary Outcome Measures

#### BREQ-3

Our comparison of the BREQ-3 data between the baseline and end of the 8-week DTx intervention revealed no significant difference in these data (Table 4).

**Table 4.** The participants’ Behavioral Regulation in Exercise Questionnaire-3 (BREQ-3) data at baseline, 4 weeks, and 8 weeks (mean and ANOVA).

		Value, mean (SD, 95% CI)	Adjusted *P* value^a^
Intrinsic motivation			
Baseline		11.2 (3.0, 10.4‐11.2)	—^b^
4 wk		11.5 (2.8, 10.7‐12.3)	.69
8 wk		11.8 (3.0, 10.9‐12.7)	.53
Integrated regulation			
Baseline		10.5 (3.2, 9.6‐11.5)	—
4 wk		10.5 (3.2, 9.5‐11.4)	.95
8 wk		10.6 (3.4, 9.6‐11.6)	.95
Identified regulation			
Baseline		13.3 (1.5, 12.9‐13.8)	—
4 wk		13.3 (2.0, 12.7‐13.9)	.99
8 wk		13.5 (2.3, 12.9‐14.2)	.64
Introjected regulation			
Baseline		8.8 (3.3, 7.8‐9.8)	—
4 wk		8.7 (3.3, 7.7‐9.6)	.90
8 wk		8.4 (3.5, 7.3‐9.8)	.52
External regulation			
Baseline		7.1 (3.4, 6.1‐8.1)	—
4 wk		7.4 (2.6, 6.6‐8.1)	.68
8 wk		7.1 (3.0, 6.2‐7.9)	.82
Amotivation			
Baseline		4.8 (2.0, 4.2‐5.4)	—
4 wk		4.8 (2.0, 4.2‐5.4)	.88
8 wk		4.9 (2.3, 4.2‐5.6)	.85

a Adjusted *P* values represent comparisons with baseline.

b Not applicable.

#### Adverse Events in This Study

No adverse events such as falls occurred.

#### Adherence to the Study

The average LT completion rate was 93% (52.1/56 days, 95% CI 50.4‐53.9), demonstrating high adherence to the smartphone-based DTx. Of the 47 participants, 46 continued using the app until the end of the study period, for a retention rate of 97%. Only 1 participant abandoned the app before the end of the study period.

## Discussion

### Principal Results

We conducted this study to determine whether the use of DTx via a smartphone app can induce behavioral change and contribute to the prevention or improvement of LS among middle-aged and older adults. To our knowledge, this is the first study assessing the efficacy of DTx for both modifying exercise behavior and managing LS. It has been reported that continued LT for a 6-month period shortened (ie, improved) participants’ TUG times [23] and that practicing 1-leg standing prevents falls [27]. It was also observed that the performance of squats enhanced the muscle strength and functional capacity of older women [28]. Another study revealed that among older adults with stage 1 LS, those who underwent LT and aerobic exercise for 8 weeks showed a significant increase in grip strength from before to after the intervention compared to those who underwent aerobic exercise alone for 8 weeks [29]. Based on this evidence, we selected LT to target lower-limb strength and balance, both of which are essential for ambulation and daily functioning.

Few studies have attempted home-based LT interventions. Aoki et al [30] reported that a 3-month program with 3×/week phone calls significantly improved their study participants’ physical function and health-related quality of life; they also described an 85.4% adherence rate for the program. Similarly, Ito et al [31] demonstrated the achievement of improved physical function through regular phone-based guidance in a preventive care setting. Although such prior studies demonstrated the efficacy of home-based LT, they were highly dependent on human resources. In contrast, our findings indicate that an app-based intervention can markedly reduce this burden, providing a scalable approach to LS management.

Our participants’ motor function was assessed by the TUG test, which showed a significant 1.5-second mean improvement post intervention. Although this change did not reach our study’s predefined MCID of 3 seconds, the mean postintervention TUG time was 7.5 seconds, which is below the average TUG time between the ages of 40 and 59 years [32]. Furthermore, the minimum detectable change for patients with knee osteoarthritis aged 45 to 70 years has been reported to be 1.10 to 1.14 [33]. The results of this study suggest that the observed changes are clinical rather than measurement errors. Considering that the MCID of 3 seconds demonstrated in previous studies was calculated from TUG scores before and after invasive procedures such as lumbar discectomy or lumbar decompression surgery [25], the results obtained in this study through a noninvasive intervention may indicate clinically meaningful improvements related to real-world activities.

In Japan, LS corresponds to the musculoskeletal ambulation disability symptom complex (MADS), which is covered by national health insurance. A TUG time ≥11 seconds is one diagnostic criterion for MADS. Notably, all participants (7/47, 14.8%) with baseline TUG times (≥11 s) improved to below this threshold after the DTx intervention. Moreover, those with longer baseline TUG times showed greater improvement. These findings suggest that our smartphone app may have potential to improve mobility for high-risk individuals meeting the MADS criteria and eligible for outpatient rehabilitation. In this study, the sample size was designed based on the MCID of TUG, and with only 7 cases exceeding TUG 11 seconds, stratified analysis proved challenging. To optimize this intervention, future research incorporating stratified analysis with a larger sample size is required.

The results of the present DTx were further supported by significant improvements in another primary outcome, the GLFS-25. After the 8-week intervention, GLFS-25 scores were markedly reduced compared with baseline. By the end of the program, 29 of 47 (62%) participants improved by at least 1 LS stage, and 16 (34%) no longer met the diagnostic criteria for LS. Among the 10 participants initially classified as stage 3, 7 improved to stage 2, suggesting potential efficacy even in advanced cases. These findings highlight the therapeutic potential of this DTx for LS management.

The effect of DTx on individuals’ exercise behavior has been assessed based on SDT [22,34]. Although our analyses of BREQ-3 results revealed no significant changes across motivational indices, some meaningful trends emerged. The number of participants reporting increased “external regulation” (a BREQ-3 subitem) remained unchanged, whereas the subitem “intrinsic motivation” and the relative autonomy index [35] tended to improve at 8 weeks compared to the baseline. These trends suggest that extended intervention may enhance participants’ autonomous motivation, warranting further investigation.

In this study, all participants received an exercise program based on LT without individualization. The exercise program demonstrated a favorable continuation rate, and no increase in amotivation or external regulation was observed on the BREQ-3; consequently, the exercise intensity is considered to have been appropriate.

### Limitations

This study has several limitations. It was a proof-of-concept investigation without a control group and involved a short observation period. Participants were likely to have high baseline health interest, introducing potential selection bias. Future studies should therefore use a randomized controlled design with a larger sample size, longer follow-up, and a control group receiving only verbal exercise instruction. In addition, the study intervention was a standardized LT protocol, which may not have been sufficiently tailored to the individual physical conditions and needs of each participant. The primary focus of this app’s functionality was on the remote management of participants’ exercise habits. However, had it been able to assess each participant’s individual exercise capacity and implement more personalized exercise programs, the improvement in exercise capacity could have been greater. Further consideration is required regarding the optimization of the app’s functionality in this regard. Moreover, the intervention did not produce a clear behavioral change in exercise habits. Without sustained behavioral modification, the long-term outcomes of app-based interventions may diminish. Integrating behavioral science–based strategies will be important to maintain user engagement and therapeutic efficacy. Finally, there may have been a measurement error in the evaluation of our participants’ TUG times. A related measurement error of 0.22 sec has been reported [36]. The measurement error in a TUG assessment is greater in frail older adults, and the longer it takes to perform the TUG test, the greater the variability is [37].

### Conclusions

The results of our analyses demonstrate that an 8-week LT intervention using DTx for middle-aged and older adults significantly improved their TUG and GLFS-25 scores. Medical staff supervised the participants’ use of the smartphone DTx, and no increase in amotivation to exercise or refusal to exercise was observed. Our findings will support the prevention and amelioration of LS in middle-aged and older adults, and the establishment of better exercise habits in this population.

## Abbreviations

BREQ-3: Behavioral Regulation in Exercise Questionnaire-3
DTx: digital therapeutics
GLFS-25: 25-question Geriatric Locomotive Function Scale
JOA: Japanese Orthopaedic Association
LS: locomotive syndrome
LSC: locomotive syndrome check
LT: locomotive training
MADS: musculoskeletal ambulation disability symptom complex
MCID: minimal clinically important difference
SDT: self-determination theory
TUG: Timed Up and Go
